# A Bird’s-Eye View of Chronic Unilateral Conjunctivitis: Remember about *Chlamydia psittaci*

**DOI:** 10.3390/microorganisms7050118

**Published:** 2019-04-30

**Authors:** Stien Vandendriessche, Joanna Rybarczyk, Pieter-Paul Schauwvlieghe, Geraldine Accou, Anne-Marie Van den Abeele, Daisy Vanrompay

**Affiliations:** 1 Department of Laboratory Medicine, Medical Microbiology, AZ Sint-Lucas Ghent, 9000 Ghent, Belgium; Anne-Marie.VanDenAbeele@AZSTLUCAS.BE; 2 Department of Laboratory Medicine, Medical Microbiology, Ghent University Hospital, 9000 Ghent, Belgium; 3 Department of Molecular Biotechnology, Faculty of Bioscience Engineering, Ghent University, 9000 Ghent, Belgium; Daisy.Vanrompay@UGent.be; 4 Department of Ophthalmology, AZ Sint-Lucas Ghent, 9000 Ghent, Belgium; pieterpaul.schauwvlieghe@ugent.be; 5 Department of Ophthalmology, Ghent University Hospital, Ghent University, 9000 Ghent, Belgium; geraldine.accou@gmail.com

**Keywords:** Chlamydia, Chlamydia psittaci, ocular infection, One Health, zoonosis

## Abstract

*Chlamydia psittaci* causes psittacosis in humans, mainly in people in contact with birds in either the setting of occupational or companion bird exposure. Infection is associated with a range of clinical manifestations from asymptomatic infection to severe atypical pneumonia and systemic disease. *C. psittaci* has also been associated with ocular adnexal lymphoma in human patients. The current paper describes successful doxycycline treatment of a male patient suffering from *C. psittaci* chronic unilateral conjunctivitis, most probably linked to the visit of a South African wildlife reserve. Increased awareness among general and occupational physicians, ophthalmologists, clinicians, and the public on the potential of *C. psittaci* to cause ocular infection is needed.

## 1. Case Description

A 57 year old Caucasian man with a blank past medical history presented at the eye clinic in 2016 with a history of a unilateral swollen eyelid and red right eye. His medical complaints started during a 10-day trip to South Africa, two days after visiting False Bay. Initially, the patient was treated in South Africa by a local general practitioner with oral azithromycin 500 mg for 3 days together with topical dexamethasone/tobramycin eye drops. After an initial improvement, 14 days later conjunctivitis symptoms flared up severely when skiing in Switzerland. Upon return to his home country, the patient consulted the eye clinic.

## 2. Medical Examination and Treatment

*Day 1.* Clinical examination showed a best-corrected visual acuity of 6/7.5 for the right eye and 6/6 for the left eye. On slit-lamp examination, primary follicles in the fornix with conjunctival hyperemia were observed. Corneal punctate epithelial keratopathy was not observed, and Tyndall effect and white blood cells were absent. The intra-ocular pressure and fundoscopy were without any abnormalities. A swab (E-swab, Liquid Amies, Copan, Italy) of the lower tarsal conjunctivae was taken. Additionally, a sample from a crust on the eye was taken. Bacterial culture of the individual ocular samples and molecular detection for *Chlamydia trachomatis/Neisseria gonorrhoeae* (BD MAX CT/GC/TV, Becton Dickinson, Franklin Lakes, NJ, USA), Herpes simplex (HSV)/Varicella Zoster (BD MAX HSV1/HSV2/VZV, Becton Dickinson, Franklin Lakes, NJ, USA), and Epstein Barr virus (EBV) on the pooled ocular sample [1] were performed. Treatment with aciclovir 800 mg, 5 times per day, was started. 

*Day 5.* The patient returned after five days with deteriorated vision and worsening of the redness of his eye. The best-corrected visual acuity was 6/6 in both eyes. At that point, slit-lamp examination showed new ulcerated lesions at the eyelid margin. Blood analysis showed no abnormalities, apart from a slightly elevated C-reactive protein (22 mg/dl). Viral/bacterial serology was negative for Cytomegalovirus, HSV, and *Borrelia burgdorferi*; and positive for EBV and Varicella Zoster (VZV). With all the above microbiological detection methods remaining negative, acyclovir treatment was discontinued.

### One Month after the First Visit

Symptoms did not disappear, and the patient consulted two other ophthalmologists and ultimately, an infectious disease physician at a travel clinic in the following weeks. A second course of 6 days azithromycin, combined with fluoroquinolone (ofloxacin) eye drops showed no improvement. At that point the patient also mentioned suffering from influenza-like symptoms (arthralgia and myalgia) at night, albeit without fever or respiratory distress. One month after the first visit, he presented back at the original eye clinic. In consultation with the infectiologist and following a thorough travel anamnesis, the patient reported to have visited a wildlife reserve in Boulders Beach, South Africa where birds and African penguins were present. As such, the possibility of conjunctivitis caused by *Chlamydia psittaci* was taken into consideration. Supplementary testing on the pooled ocular sample taken during the first visit (ocular E-swab in Liquid Amies transport medium and a sample from a crust on the eye) using a *C. psittaci*-specific nested PCR [2] revealed a positive result. A genotype-specific real-time PCR targeting the gene encoding the outer membrane protein A (*ompA*), confirmed the presence of *C. psittaci* genotype B [3]. However, a serological response to *C. psittaci* (Anti-Chlamydia HP Euroline-WB Ig, Euroimmun, Lubeck, Germany) could not be detected. A chest radiograph to exclude signs of consolidation, a frequent manifestation of psittacosis, was negative. The patient was successfully treated with doxycycline 100 mg twice daily for two weeks, after which all symptoms were resolved. For publication of case study descriptions, an informed consent was signed by the patient. 

## 3. Discussion

One of the most typical pathogens associated with chronic unilateral conjunctivitis is *C. trachomatis,* but as demonstrated by the present case, *C. psittaci* should also be taken into consideration as a causative agent. Members of the genus *Chlamydiaceae* are obligate intracellular gram-negative bacteria that are able to cause a variety of acute and chronic infections in humans, other mammals, and birds. While *C. trachomatis* and *C. pneumoniae* are typical human pathogens, other *Chlamydia* species predominantly cause zoonotic infections [4]. 

Birds are the main reservoir for *C. psittaci* [4]. Avian chlamydiosis caused by *C. psittaci* has been known for centuries. *C. psittaci* infections are widespread throughout the world. Based on isolation, antigen detection, and serology, *C. psittaci* can infect more than 450 bird species from at least 30 different orders [5]. The avian host range is probably even broader when diagnosis is made by PCR, a more sensitive and specific diagnostic technique that has replaced culture as the gold standard for *Chlamydia* diagnosis. Consequently, human infection caused by *C. psittaci* (predominantly psittacosis and pneumonia) is frequently linked with occupational exposure to infected/colonized birds. Transmission from animals to humans occurs through inhalation of contaminated aerosols from urine/feces, respiratory, and eye secretions, for example during cage cleaning or due to bird bites [4]. However, as suspected also by the present case, transient exposure, for instance by visiting birds/penguins in wildlife reserves, can also cause chlamydial conjunctivitis. 

Non-chlamydial trachomatis conjunctivitis is rarely reported, and data regarding the prevalence of *C. psittaci* in conjunctivitis cases are rare. One study performed in the United States of America in 1998 found that 20% of patients with chronic conjunctivitis (> 1 month’s duration) who tested positive for chlamydial lipopolysaccharides (genus-specific LPS) but negative for *C. trachomatis*-specific major outer-membrane protein (MOMP), had evidence of *C. psittaci* DNA in conjunctival samples [6]. Two surveys provide a clue on the prevalence of *C. psittaci* in trachoma, the chronic form of Chlamydia ocular infections in Nepal, a trachoma endemic region. *C. psittaci* was detected in monomicrobial as well as in mixed *Chlamydiaceae* infections, in a prevalence ranging from 10–37% [7,8]. Taken together, these data suggest that ocular infections other than *C. trachomatis* are probably more common than expected. Distinction between *C. psittaci* and *C. trachomatis* is especially relevant since longer treatment intervals might be required for *C. psittaci* ocular infections. While a single dose of azithromycin 1 g is considered adequate for the treatment of *C. trachomatis* conjunctivitis [9], longer treatment seems to be necessary for conjunctivitis caused by *C. psittaci* [6]. Indeed, this patient received multiple oral (two treatment courses with azithromycin for 3 and 6 days, respectively) and local (tobramycin/dexamethasone and ofloxacin eye drops) antibiotic courses, before resolution of symptoms following a 2-week course of doxycycline. 

Importantly, *C. psittaci* conjunctivitis should not be neglected as chronic ocular *C. psittaci* infections have been associated with ocular adnexal marginal zone lymphoma (OAMZL) [10,11]. Ferreri et al. (2012) presented the results of an international phase II trial on the eradication of *C. psittaci* using doxycycline as first-line targeted therapy for ocular adnexae lymphoma [12]. Upfront doxycycline treatment (100 mg orally twice daily for 3 weeks) proved to be a rational and active treatment for patients with stage I *C. psittaci* OAMZL. Lymphoma regression was consequent to *C. psittaci* eradication, monitored on conjunctival and blood samples. 

*C. psittaci* ocular infection is probably underdiagnosed and therefore underestimated. Currently, awareness of *C. psittaci* ocular infection and its clinical presentations is low among general and occupational physicians, ophthalmologists, and clinicians. Most of the conjunctivitis patients are managed in primary care, by general practitioners, and current professional guidelines do not recommend microbial testing for patients with conjunctivitis, and so the patients are treated, but the causative agent remains unknown. 

Several *C. psittaci* diagnostic tests are available. Culture has several well-known disadvantages, for instance viable organisms and BSL3 are required, and is therefore seldom performed. Zoonotic transmission is most often diagnosed by serology. However, *C. psittaci* serological tests show cross-reactivity with other chlamydial species and they don’t allow source tracing. In addition, it may take several weeks to develop a detectable serological response. The patient presented here was seronegative in the presence of clinical disease and positive by *C. psittaci* PCR. False seronegative results are possible during the acute phase of psittacosis [13,14]. Also, a serological response can be negatively influenced by antibiotic treatment and by genetic variations in Toll-like and/or NOD-like receptors leading to inadequate recognition of *Chlamydia* by the hosts immune system [15]. Thus, clinical decision-making concerning patient management based on serology is unwise and should be avoided. An increasing number of medical microbiological laboratories are therefore switching to real-time qPCR. Real-time qPCR is rapid, more sensitive, and more specific than serology [16]. An additional advantage of real-time qPCR, as also demonstrated here, is that DNA used for the detection of the pathogen, can also be used for *ompA* genotyping by use of genotype (A to F and E/B) specific probes [3]. Molecular typing supports source tracing and possible treatment of the infection source which was not possible in this case as the source most probably originated from the South African wildlife reserve. Unfortunately, *C. psittaci* PCR is not yet routinely implemented as National laboratory testing regulations often require clinical laboratories to be certificated before they can accept clinical samples for *C. psittaci* testing by nucleic acid amplification tests (for instance, psittacosis real-time PCR is in the U.S. not Clinical Laboratory Improvement Amended (CLIA)-validated; https://www.fda.gov/) [17]. Another, perhaps even more important bottleneck for going to nucleic acid amplification tests, is the fact that national health insurances reimburse serology, but nucleic acid amplification tests are not always reimbursed.

In conclusion, in patients with a chronic follicular conjunctivitis that does not respond well to topical or short oral antibiotic therapy and who report a history of occupational/recreational exposure to birds (including penguins), a non-chlamydial trachomatis conjunctivitis should be considered. Diagnosis is to be performed by *C. psittaci* specific PCR directly on the ocular sample, as *C. psittaci* serology can remain negative. A good collaboration between ophthalmologists and the laboratory is essential to make a proper diagnosis.
